# Triple-Gene Overexpression of the AcrA-AcrB-TolC Transporter System in *Synechocystis* sp. PCC 6803 Contributes to a Higher Secretion of Free Fatty Acids in Response to Nitrogen Shortage and Salt Stress

**DOI:** 10.3390/ijms252212131

**Published:** 2024-11-12

**Authors:** Kamonchanock Eungrasamee, Peter Lindblad, Saowarath Jantaro

**Affiliations:** 1 Laboratory of Cyanobacterial Biotechnology, Department of Biochemistry, Faculty of Science, Chulalongkorn University, Bangkok 10330, Thailand; 2 Microbial Chemistry, Department of Chemistry—Ångström, Uppsala University, Box 523, SE-75120 Uppsala, Sweden

**Keywords:** *Synechocystis* sp. PCC 6803, lipids, free fatty acids, transportation system

## Abstract

One important aspect of cyanobacterial homoeostasis is reducing the toxicity of excess free fatty acids (FFAs) generated in the cells by means of both secreting these into the medium and recycling them toward membrane lipid synthesis. In this study, the cyanobacterium *Synechocystis* sp. PCC 6803 served to implement the overexpression of native genes of the transportation system. Specifically, we worked with the Sll0180-Slr2131-Slr1270 homologs of *Escherichia coli* AcrA-AcrB-TolC, respectively, to create single- and triple-overexpressing strains of OA, OB, OC, and OABC. Remarkably, the OABC strain that triply overexpressed the sll0180_slr2131_slr1270 genes acquired a significant amount of intracellular lipids, up to 23.5% of dry cell weight, under the normal condition. Nitrogen-deficient stress undoubtedly raised extracellular FFAs and intracellular lipids in overexpressing strains, especially in the OABC strain, which exhibited 33.9% and 41.5% of dry cell weight, respectively. During the first 5 days of treatment, salt stress at 256 mM significantly increased the FFA efflux, notably for the OB strain, but had no effect on intracellular lipids. It is noteworthy that the OA and OABC strains outperformed all other strains in terms of growth throughout the 16 days of nitrogen shortage. Furthermore, in comparison to the wild-type control, all the overexpressing strains exhibited a considerable increase in carotenoid accumulation. Thus, our results point to the effective role of the sll0180_slr2131_slr1270 transportation system in facilitating FFA secretion, especially in response to environmental stressors.

## 1. Introduction

Regarding the action on clean energy, climate change, and life below water in the Sustainable Development Goals (SDGs), cyanobacteria are one of the main bioproducers of biofuels and valuable biocompounds, such as glycogen, alka(e)ne, polyhydroxybutyrate (PHB), fatty acid alcohol, lipids, and free fatty acids (FFAs), from atmospheric CO_2_ fixation during photosynthesis [1,2,3,4,5,6,7,8,9]. In terms of biomass, cyanobacterial cells retain significant amounts of free fatty acids (FFAs) and intracellular lipids in their membrane system, which includes the outer membrane, plasma membrane, and thylakoid membrane [10]. The transesterification method can then be used to transform free fatty acids into biodiesel [11,12]. Consequently, the production of lipids and FFAs by cyanobacteria has garnered interest recently, along with their pharmaceutical, food, and cosmetic applications [13]. Certain fatty acids, such as lauric acid (C12:0) and myristic acid (C14:0), can protect rats from developing prostatic hyperplasia [14].

**Figure 1:**
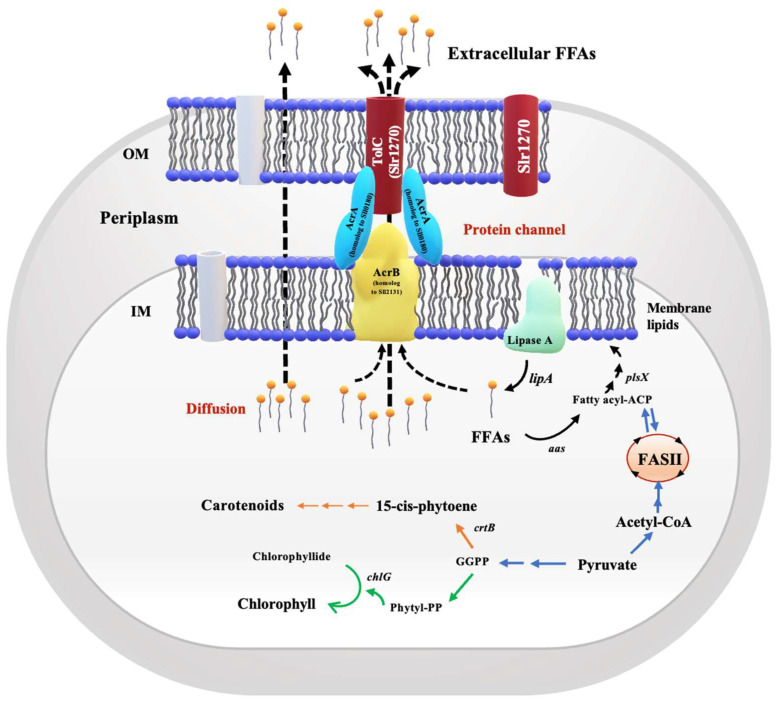
An overview of extracellular FFA secretion, which includes diffusion and protein channels, such as the ABC transporter containing the Sll0180, Slr2131, and Slr1270 proteins, which are homologs of *Escherichia coli* AcrA, AcrB, and TolC, respectively, and are located on the outer membrane (OM) and inner membrane (IM). Intracellular FFAs, generated by membrane hydrolysis via lipase A catalysis encoded by the *lipA* gene, can be recycled through membrane lipid synthesis by initially converting to a substrate fatty acyl–acyl carrier protein (fatty acyl–ACP) via the *aas* gene encoding fatty acyl–ACP synthetase (AAS). Then, fatty acyl–ACPs are subsequently combined with the glycerol backbone of glycerol-3-phosphate via putative phosphate acyltransferases, encoded by the *plsX* gene, and several reactions to synthesize membrane lipids. Acetyl–CoA, a crucial intermediate, is the primary precursor for lipid synthesis and is mostly derived from pyruvate. For the neighboring pathway, pyruvate partly flows through the 2-C-methyl-d-erythritol-4-phosphate (MEP) pathway to generate geranylgeranyl pyrophosphate (GGPP), the precursor to many compounds, such as carotenoids, which is converted into 15-*cis*-phytoene via phytoene synthase (CrtB; encoded by the *crtB* gene), or converted into chlorophyll by being converted to phytyl–PP and, together with chlorophyllide, via the activity of chlorophyll synthase encoded by the *chlG* gene. The dashed arrow indicates a possible path of FFA movement.

Cyanobacteria synthesize lipids and FFAs through a series of stages in Fatty Acid System II (FASII), beginning with acetyl–CoA and ending with acyl–ACP, also known as fatty acyl–acyl carrier protein (or fatty acyl–ACP), which is a crucial precursor to synthesizing membrane lipids (Figure 1). The *lipA* gene, encoded by the *sll1969* gene found on the membrane, can hydrolyze membrane lipids to free fatty acids (FFAs) [15,16]. Intracellular FFAs are generated as a result of this breakdown and are mostly recycled for lipid synthesis by acyl–ACP synthetase (AAS), which is encoded by the *slr1609* or *aas* gene for conversion back to acyl–ACP in *Synechocystis* sp. PCC 6803 [2,17]. In addition, either randomly diffusing excessive free fatty acids (FFAs) across the cell membrane or turning out un-ionized FFAs across membrane protein channels, such as efflux transmembrane transporters, might result in the rapid removal of the excess FFAs’ toxicity [15,18,19]. Due to their structure, which enables them to disrupt thylakoid and cell membranes as well as destabilize essential membrane proteins, cyanobacteria have restrictions on their intracellular free fatty acid levels [20,21,22]. Nonetheless, the benefits of cyanobacteria-secreting FFAs are apparently exceptionally encouraging for intracellular homeostatic balance. This is because the products are readily collected, which lowers the expense and time required for FFA recovery and extraction [23].

Overexpression of the *lipA* gene, which encodes the lipase A enzyme, and the disruption of fatty acyl–ACP production through *aas* knockout in the fatty-acid-recycling process in *Synechocystis* sp. PCC 6803 successfully unlocked the restricted production with sustained growth to attain a greater secretion of FFAs [16]. The heterologous expression of the thioesterase gene combined with weakened polar cell wall layers is also a good strategy for increasing FFA secretion [24]. Furthermore, free fatty acid secretion was dramatically promoted by the synergistic effect of doubly knocking out the *aas* gene with the *sll1951* gene encoding the surface (S) layer protein in *Synechocystis* sp. PCC 6803 after induction with a BG_11_ medium lacking nitrogen by about 6 times higher than in the wild type [25]. Nevertheless, earlier studies have established that S-layer monomeric proteins are produced in the cytosol [26] and then released from cells through the trimeric outer membrane protein Slr1270, a homolog of the *Escherichia coli* TolC found in *Synechocystis* sp. PCC 6803 [27,28]. This TolC is part of the Type I secretion system [29], which interacts with two other proteins in the plasma membrane, the membrane fusion protein Sll1181 and an ABC transporter Sll1180, to provide a direct channel for the export of S-layer proteins or other metabolites [30]. Specifically, it was discovered that the TolC duct protein works with AcrA and AcrB to function as a multidrug efflux pump in *E. coli* to achieve antibiotic tolerance [31,32,33]. Recent substantial findings in *Synechocystis* sp. PCC 6803 logically illustrated how distinct TolC functions, alongside interaction with distinct transporters, affect how the cell responds to stress or toxicity in terms of homeostasis [34]. The system of Sll0180-Sll2131-TolC transportation in *Synechocystis* sp. PCC 6803 was reported to substantially contribute to not only chloramphenicol tolerance but also free fatty acid secretion [35]. To clarify the function of the Slr2131 and Sll0180 homologs with AcrB and AcrA, respectively, from *E. coli*, the significant decrease in FFA secretion found in both *Synechocystis* sp. PCC 6803 mutants lacking the *slr2131* and *sll0180* genes was restored by introducing the heterologous *E. coli acrB* and *acrA* genes. Therefore, it was considered that Sll0180 and Slr2131 are possibly involved in FFA efflux [35].

In accordance with prior research on application and sustainable production, we engineered four strains of *Synechocystis* sp. PCC 6803 to enhance FFA secretion. These strains were genetically optimized by overexpressing *sll0180* (a Sll0180 homolog of AcrA), *slr2131* (a Slr2131 homolog of AcrB), *slr1270* (a Slr1270 homolog of TolC), and triple *acrA_acrB_tolC* genes, referred to as OA, OB, OC, and OABC, respectively. A higher growth level under nitrogen-deprived conditions was certainly found in overexpressing strains, in particular OA, OC, and OABC, whereas the NaCl treatment at 1.5% (*w*/*v*) or 256 mM did not show any changes among all strains. Unexpectedly compared to the wild-type control, overexpressing strains were demonstrated to have a much greater production of free fatty acids (FFAs), particularly on day 5 of cell growth.

## 2. Results

### 2.1. Overexpressions of Native sll0180, slr2131, and slr1270 Genes in Synechocystis sp. PCC 6803

**Figure 2:**
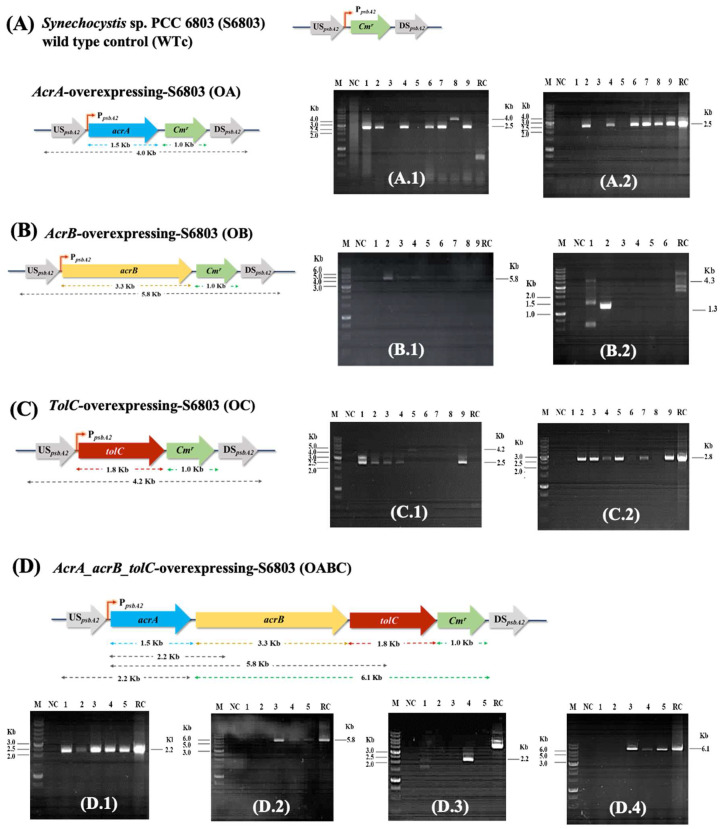
The genomic maps of engineered *Synechocystis* sp. PCC 6803 strains, including wild-type control (WTc), OA (**A**), OB (**B**), OC (**C**), and OABC (**D**). The confirmation of inserted gene location was verified by PCR analysis using specific pairs of primers (Supplementary information, Table S1). All engineered strains were constructed by overexpressing each native gene in the WTc. The double homologous recombination of each gene occurred between the conserved sequences of *psbA2* gene in WT (**A**–**D**). For WTc (**A**), the *Cm^r^* gene fragment was introduced to WT genome at the *psbA2* region. For the OA strain (**A**), the *sll0180-Cm^r^* gene fragment was introduced into the WT genome; (**A.1**,**A.2**) Lane M: 1 Kb DNA ladder (SibEnzyme^®^, SibEnzyme US LLC, Franclaire Drive, West Roxbury, MA, USA); (**A.1**) PCR products using UUSpsbA2 and DDSpsbA2 primers with an expected size of 4.0 Kb for positive clone and (**A.2**) PCR products using Sll0180_F and Cm_R primers with an expected size of 2.5 Kb for positive clone; Lane NC: negative control (without template), Lanes 1–9: clones no. 1–9, Lane RC (recombinant plasmids): positive control. A positive clone no. 8 was taken for further experiment. For the OB strain (**B**), the *slr2131-Cm^r^* gene fragment was introduced into the WT genome, (**B.1**,**B.2**) Lane M: 1 Kb DNA ladder; (**B.1**) PCR products using UUSpsbA2 and DDSpsbA2 primers with an expected size of 5.8 Kb for positive clone, and (**B.2**) PCR products using Slr2131_F and Cm_R primers with an expected size of 4.3 Kb for positive clone; Lane NC: negative control (without template), Lanes 1–6: clones no. 1–6, Lane RC: positive control. A positive clone no. 1 was taken for further experiment. For the OC strain (**C**), the *slr1270-Cm^r^* gene fragment was introduced into the WT genome; (**C.1**,**C.2**) Lane M: 1 Kb DNA ladder; (**C.1**) PCR products using UUSpsbA2 and DDSpsbA2 primers with an expected size of 4.2 Kb for positive clone, and (**C.2**) PCR products using Slr1270_F and Cm_R primers with an expected size of 2.8 Kb for positive clone; Lane NC: negative control (without template), Lanes 1–9: clones no. 1–9, Lane RC: positive control. A positive clone no. 5 was taken for further experiment. For the OABC strain (**D**), the *sll0180-slr2131-slr1270-Cm^r^* gene fragment was introduced into the WT genome; (**D.1**–**D.4**) Lane M: 1 Kb DNA ladder; (**D.1**) PCR products using Sll0180_F and RTacrB_R380 primers with an expected size of 2.2 Kb for positive clone, (**D.2**) PCR products using Sll0180_F and RTtolC_R480 primers with an expected size of 5.8 Kb for positive clone, (**D.3**) PCR products using UUSpsbA2 and Sll0180_R primers with an expected size of 2.2 Kb for positive clone, (**D.4**) PCR products using Slr2131_F and Cm_R primers with an expected size of 6.1 Kb for positive clone; Lane NC: negative control (without template), Lanes 1–5: clones no. 1–5, Lane RC: positive control. A positive clone no. 4 was taken for further experiment.

Prior to being independently transformed into the host *Synechocystis* sp. PCC 6803 wild-type cells, the recombinant pECm_acrA, pECm_acrB, pECm_tolC, and pECm_acrA/acrB/tolC plasmids were created (Table 1). The surviving transformants were cultured on a BG_11_ agar containing 35 µg/mL of chloramphenicol antibiotic for a few weeks. After being randomly selected, they underwent PCR analysis with certain primer pairs (Supplementary Information Table S1) to determine the location and segregation of the genes (Figure 2A–D).

All the engineered strains were then constructed, as a consequence, via double homologous recombination by crossing over the DNA fragments between the flanking *psbA2* sequences located on the pEERM vector. The *psbA2* gene in the *Synechocystis* sp. PCC 6803 wild-type (WT) strain was replaced with a *Cm^r^* cassette to form the wild-type control (WTc) strain (Figure 2A). The PCR products with UUSpsbA2 and DDSpsbA2 primers confirmed the expected sizes of about 4.0, 5.8, and 4.2 Kb, respectively, in OA, OB, and OC strains (Figure 2(A.1,B.1,C.1)). No bands were seen in Lane RC when the recombinant plasmids pECm_acrA (Figure 2(A.1)), pECm_acrB (Figure 2(B.1)), and pECm_tolC (Figure 2(C.1)) were used as DNA templates regarding the outer-flanking-region design of the UUSpsbA2/DDSpsbA2 primers on the *Synechocystis*’s genome. And the PCR products with Sll0180_F and Cm_R primers confirmed the correct size of 2.5 Kb fragment (Figure 2(A.2)). Then, the positive transformant for OA strain was clone no. 8. For the OB strain, the PCR products with Slr2131_F and Cm_R primers confirmed the correct size of 4.3 Kb fragment (Figure 2(B.2)). A positive transformant, clone no. 1, for the OB strain was chosen. The OC transformant was confirmed by the PCR products, with Slr2170_F and Cm_R primers showing the correct size of 2.8 Kb (Figure 2(C.2)). A selected transformant for the OC strain was clone no. 5. On the other hand, for the OABC strain, four pairs of primers were performed in the PCR reaction (Supplementary Information Table S1): a pair of Sll0180_F and RTacrB_R380 primers giving a 2.2 Kb fragment (Figure 2(D.1)), a pair of Sll0180_F and RTtolC_R480 primers giving a 5.8 Kb fragment (Figure 2(D.2)), a pair of UUSpsbA2 and Sll0180_R primers giving a 2.2 Kb fragment (Figure 2(D.3)), and a pair of Slr2131_F and Cm_R primers giving a 6.1 Kb fragment (Figure 2(D.4)). Clone no. 4 was a positive transformant for OABC strain. For the Lane RC of Figure 2(D.3), regarding the outer-flanking-region design of the UUSpsbA2/Sll0180_R primers on the genome of *Synechocystis*, no band was seen when the recombinant plasmid pECm_ acrA/acrB/tolC was employed as the DNA template.

### 2.2. Cell Growth, Intracellular Pigment Contents, and O_2_ Evolution Rates Under Stress Conditions

**Figure 3:**
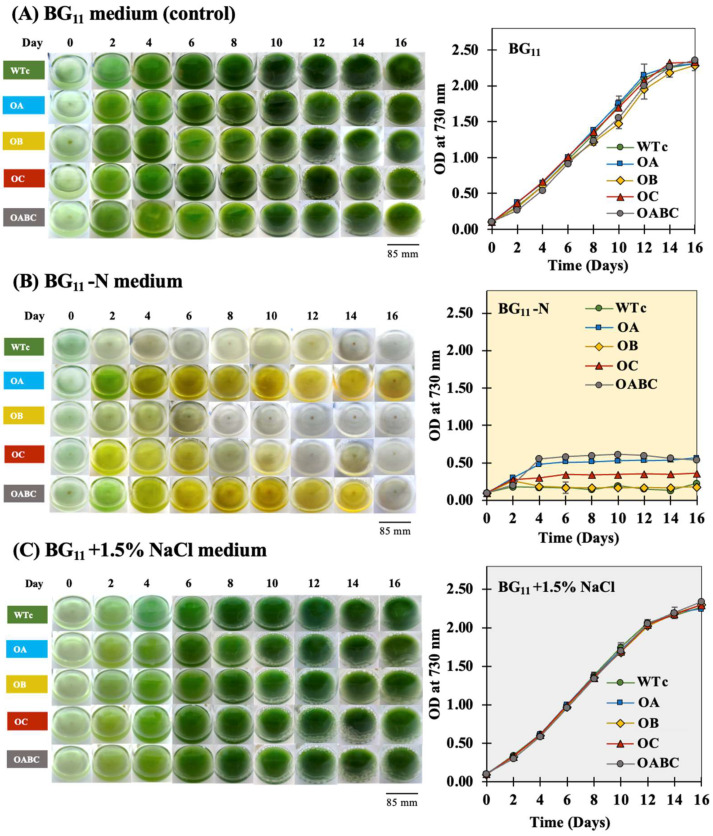
Images of cell-cultured 250 mL flasks and optical density at 730 nm (OD_730_) of *Synechocystis* sp. PCC 6803 WTc, OA, OB, OC, and OABC strains cultured under normal BG_11_ (**A**), BG_11_-N (**B**), and BG_11_+1.5% (*w*/*v*) NaCl (**C**) conditions for 16 days. The error bars represent standard deviations of means (mean ± S.D., *n* = 3).

We imposed stress on the cell growth of all engineered strains, including salt stress (1.5% NaCl, *w*/*v*) and nitrogen scarcity (BG_11_ lacking NaNO_3_), (Figure 3). The prevalence of new unsaturated fatty acid compositions in the thermal freshwater microalgae *Scenedesmus* sp. was induced by salinity at 10 g/L NaCl, with regard to how the membrane integrity responded to environmental change [37]. Furthermore, in the *Synechocystis* sp. PCC 6803 wild type and a mutant-lacking S-layer protein, nitrogen deficiency stress, which has a substantial influence on the metabolic balance of energy in cyanobacteria, dramatically stimulated both the intracellular lipid accumulation and the release of FFAs [25]. Comparing the overexpressing strains to the wild-type control, our findings demonstrated that the overexpressions of the *acrA*, *acrB*, and *tolC* genes had no negative effects on growth or developmental stages under normal growing conditions (Figure 3A), while nitrogen-deprived conditions certainly decreased cell growth during the 16 days of cultivation (Figure 3B). However, resulting from nitrogen deprivation, the OB strain had a pattern like that of the wild-type control, whereas the OA, OC, and OABC strains showed a higher OD_730_ level. On the other hand, as compared with typical BG_11_ medium, the mild salt stress of 1.5% NaCl or 256 mM prompted greater cell growth but was not harmful to any strain’s capability to grow (Figure 3C).

**Figure 4:**
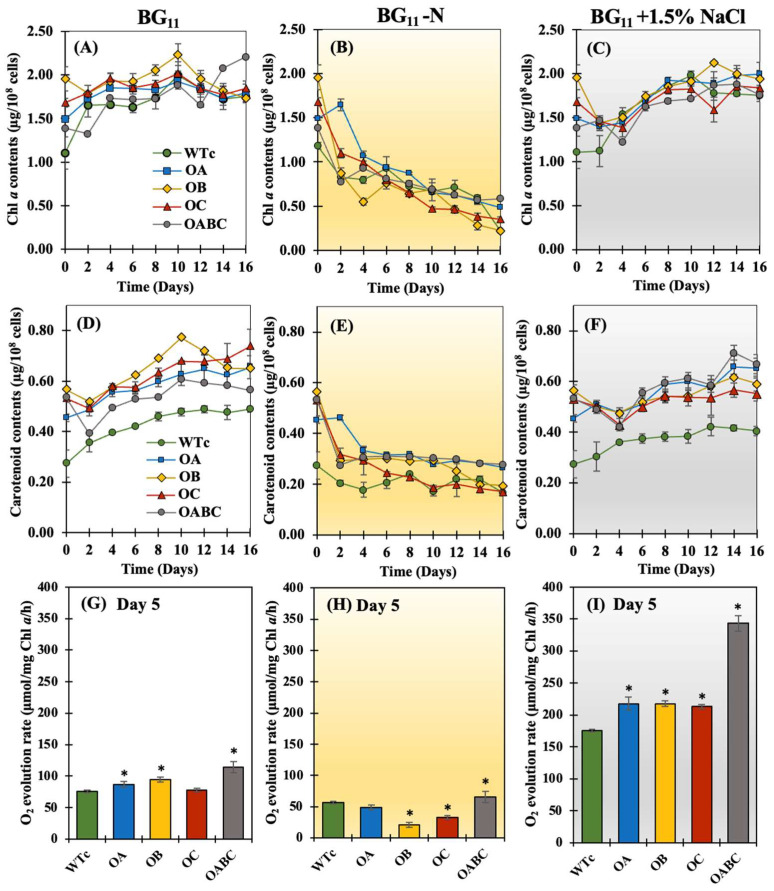
Contents of chlorophyll *a* (**A**–**C**), carotenoids (**D**–**F**), and O_2_ evolution rates (**G**–**I**) of *Synechocystis* sp. PCC 6803 WTc, OA, OB, OC, and OABC strains cultured under normal BG_11_ (**A**,**D**,**G**), BG_11_-N (**B**,**E**,**H**), and BG_11_+1.5% NaCl (**C**,**F**,**I**) conditions for 16 days. The oxygen evolution rate of all strains studied was determined at day 5 of treatment. The error bars represent standard deviations of means (mean ± S.D., *n* = 3). The statistical difference (Student’s *t*-test) between the values of WTc and engineered strain is represented by an asterisk, * *p* < 0.05.

The chlorophyll *a* contents of overexpressing strains were comparable to those of the wild-type control in all media studied (Figure 4A–C). In contrast, a certain increase in carotenoids’ accumulation occurred in overexpressing strains, which was higher than the wild-type control (WTc) under all conditions, in particular OA and OABC in BG_11_-N and BG_11_+1.5% NaCl media (Figure 4D–F). Additionally, overexpressing strains growing at log phase showed greater O_2_ evolution rates than WTc, representing cyanobacterial photosynthetic efficiency, except for the OC strain under normal BG_11_ condition (Figure 4G). It appears that the salt stress in creased all strains’ photosynthetic efficiency, especially the OABC strain (Figure 4I). Conversely, in the nitrogen-starved condition, photosynthetic efficiency was significantly reduced (Figure 4H).

### 2.3. Accumulation of Intracellular Lipids and Free Fatty Acid Secretion in Engineered Synechocystis sp. PCC 6803 Strains

**Figure 5:**
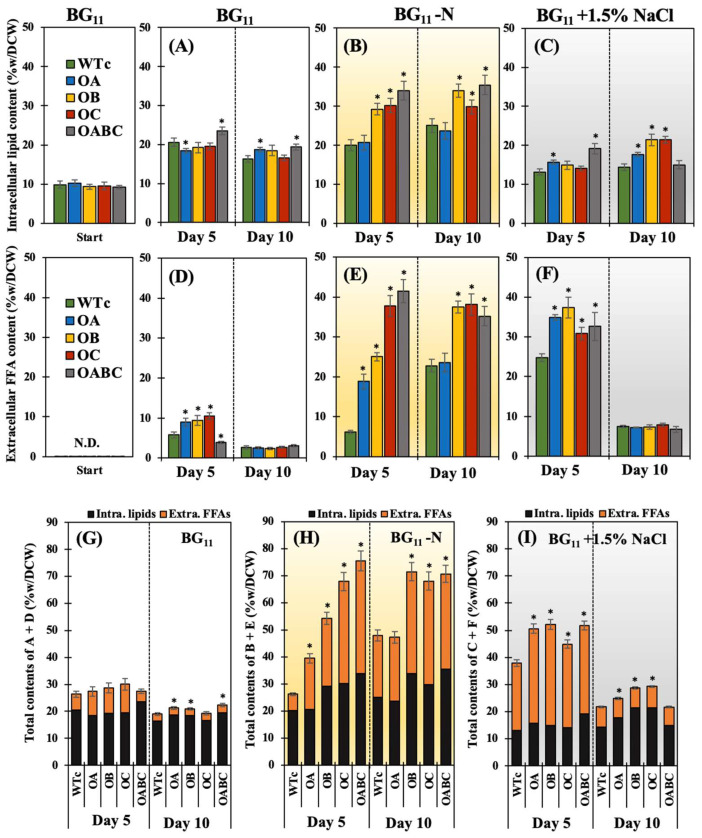
Contents of intracellular lipids (**A**–**C**), extracellular FFAs (**D**–**F**), and total contents of intracellular lipids and extracellular FFAs (**G**–**I**) of *Synechocystis* WTc, OA, OB, OC, and OABC strains cultured under normal BG_11_ (**A**,**D**,**G**), BG_11_-N (**B**,**E**,**H**), and BG_11_+1.5% NaCl (**C**,**F**,**I**) conditions at days 5 and 10 of treatment. The error bars represent standard deviations of means (mean ± S.D., *n* = 3). The statistical difference (Student’s *t*-test) between the values of WTc and the engineered strain is represented by an asterisk, * *p* < 0.05.

Stress deficient in nitrogen, as opposed to normal growth conditions and salt stress, was a remarkable inducer of intracellular lipid accumulation (Figure 5A–C). Significantly increased intracellular lipid content (%w/DCW) was produced by the OB, OC, and OABC strains at the log and late log phases of cell growth or correspondingly on days 5 and 10 (Figure 5B). Under the normal BG_11_ condition, the intracellular lipid production (mg/L) of the OA and OABC strains at day 10 was not significant (at *p* < 0.05), but Figure 5A shows a substantial change in their intracellular content (%w/DCW) depending on the varied dry cell weight (DCW) at day 10. When nitrogen was scarce, the OABC strain’s highest level of intracellular lipids was around 33–35% of dry cell weight, although the yield (mg/L) was comparable with wild-type control (Table 2). However, salt stress with a 1.5% NaCl concentration was unlikely to be a possible inducer of increased intracellular lipids. It is noteworthy that, in contrast to the WTc, the OABC strain also had higher levels of intracellular lipids under salt stress during the log phase (day 5) of growth (Figure 5C and Table 2).

**Figure 6:**
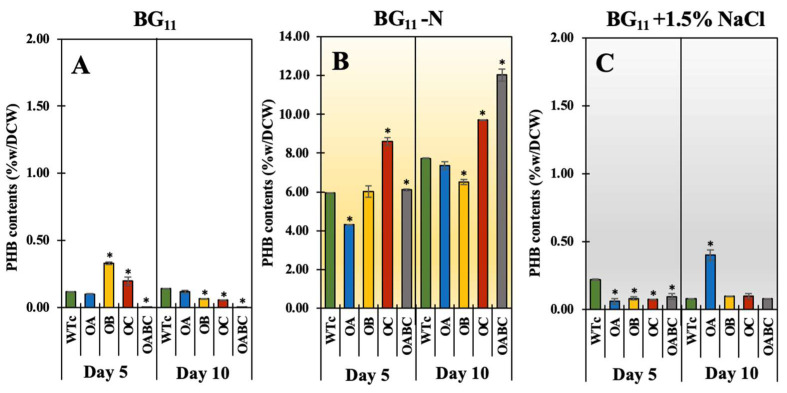
Contents of polyhydroxybutyrate (PHB) in of *Synechocystis* sp. PCC 6803 WTc, OA, OB, OC, and OABC strains cultured under normal BG_11_ (**A**), BG_11_-N (**B**), and BG_11_+1.5% NaCl (**C**) conditions at days 5 and 10 of treatment. The error bars represent standard deviations of means (mean ± S.D., *n* = 3). The statistical difference (Student’s *t*-test) between the values of WTc and the engineered strain is represented by an asterisk, * *p* < 0.05.

On the other hand, cells exposed to the nitrogen-deprived state, particularly the OB, OC, and OABC strains, appeared to preferentially secrete FFAs into the medium in comparison to cells under the normal BG_11_ condition (Figure 5D,E). Overexpressing strains, in particular OB, responded to salt stress during the log phase of cell growth by secreting a sharp amount of FFA (Figure 5F). It is crucial to note that on day 5, after the NaCl treatment, the extracellular FFA yield (mg/L) released by the overexpressing strains was greater than under other conditions during the log phase (Table 2). In terms of increased biomass, contents under salt stress as a percentage of dry cell weight were lower than contents during nitrogen deficiency. Total contents of intracellular lipids and secreted FFAs were augmented by nitrogen shortages in all strains, in particular OB, OC, and OABC (Figure 5G–I). Nevertheless, as anticipated, the lack of nitrogen had a greater effect on PHB accumulation than both salt stress and normal growth conditions, in particular OC and OABC strains (Figure 6).

### 2.4. Transcript Levels of Genes Under Nitrogen Deprivation and Salt Stress

**Figure 7:**
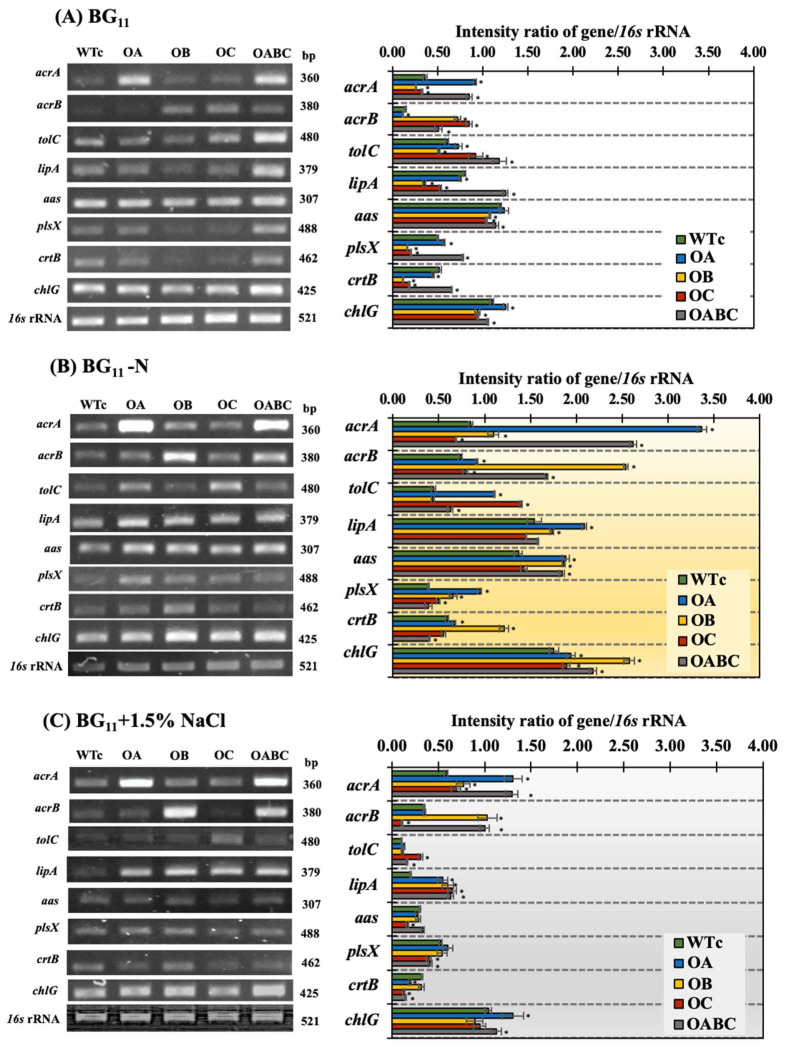
Transcript levels of *acrA*, *acrN*, *tolC*, *lipA*, *aas*, *plsX*, *crtB*, and *chlG* genes and their relative intensity ratios of each gene/*16s* rRNA of *Synechocystis* sp. PCC 6803 WTc, OA, OB, OC, and OABC strains cultured at day 5 of treatment under normal BG_11_ (**A**), BG_11_-N (**B**), and BG_11_+1.5% NaCl (**C**) conditions. The error bars represent standard deviations of means (mean ± S.D., *n* = 3). The statistical difference (Student’s *t*-test) between the values of WTc and the engineered strain is represented by an asterisk, * *p* < 0.05. The original images of RT-PCR products on a 1.5% agarose gel were shown in Supplementary Information Figures S1–S3.

**Figure 8:**
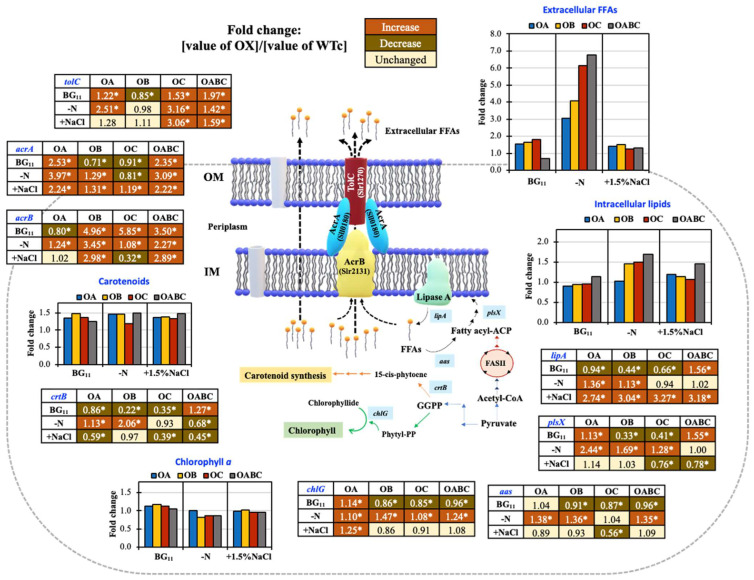
Fold changes in metabolite contents and gene transcript levels in four engineered strains compared with those in *Synechocystis* WTc. Cells were cultured in normal BG_11_, BG_11_-N, and BG_11_+1.5% NaCl media at day 5 of treatment. In each box and graph, the number and bar graph represent the fold change in that value in each engineered strain divided by that value in WTc. The statistical difference (Student’s *t*-test) between the values of WTc and the engineered strain is represented by an asterisk, * *p* < 0.05. The colors in each box of gene transcript level are as follows: orange means an increase in a fold change, brown means a decrease in a fold change, and yellow means no change.

The *acrA*, *acrB*, and *tolC* gene overexpressions were validated by RT-PCR data in all overexpressing strains exhibiting higher transcript levels compared to the wild-type control (Figure 7). During the log phase of growth, it was discovered that, in the wild-type control, the *tolC* gene had the highest transcript level under the normal condition relative to the levels of *acrA* and *acrB* transcripts (Figure 7A), while nitrogen deficiency and salt stress had influenced the lowest amount of *tolC* transcript compared with *acrA* and *acrB* transcripts (Figure 7B,C). It is vital to take into account that *tolC* overexpression could result in 5.85 times more *acrB* mRNA in the OC strain than in the WTc under the normal BG_11_ condition (Figure 7A and Figure 8).

Compared to WTc, the OABC strain had higher expression levels of the *plsX* and *lipA* genes, which are involved in membrane lipid production and membrane breakdown, respectively. The OABC strain had a greater carotenoid content than the WTc strain under the normal condition (Figure 4D), which could have been explained by an elevated *crtB* transcript level (Figure 7A). However, findings with decreased *crtB* transcript levels were not consistent with the carotenoid contents in other overexpressing strains. In addition, the *chlG* transcript levels, related to chlorophyll synthesis, were comparable among all strains. Yet another contrast to strains under the normal BG_11_ condition: nitrogen shortage (BG_11_-N) demonstrated a powerful inducer to enhance *lipA*, *aas*, and *chlG* genes in all strains (Figure 7B). The levels of *plsX* and *aas* transcripts, which are involved in the FFA-recycling cycle and membrane lipid synthesis, respectively, were elevated in the presence of nitrogen deprivation relative to WTc (Figure 8). On the other hand, in contrast to the WTc, salt stress at a concentration of 1.5% NaCl caused an increase in the *lipA* transcript, involved in the breakdown of membrane lipids, in all overexpressing strains, which was attributed to the higher secretion of FFAs (Figure 5F). Furthermore, it was noticed that the levels of *chlG* transcripts, related to chlorophyll synthesis, were greater than those of *crtB* transcripts involved in carotenoid synthesis, for all strains (Figure 7C).

## 3. Discussion

It is commonly recognized in the field that the free fatty acid (FFA)-recycling reaction and their release into the medium biologically balance the excess free fatty acids (FFAs) in cyanobacteria [16,24,25,38,39]. Free fatty acid (FFA) diffusion and channel protein membranes are the two mechanisms that allow FFA transportation [15,18]. In the absence of environmental stress, the intracellular free fatty acid may favor random diffusion across cell membranes and move itself into the medium, thus balancing intracellular metabolites. Recent findings have confirmed that weakening cyanobacterial membranes by disrupting the sll1951-gene-encoding S-layer protein efficiently enhanced the secretion of FFAs into the medium [24,25]. Once within the cells, the S-later proteins are discharged via the Slr1270 membrane protein transporter found in *Synechocystis* sp. PCC 6803, an *Escherichia coli* TolC homolog [27,28]. Given the versatility of TolC, it may be linked to a number of inner membrane complexes that facilitate the transport of a range of substrates involved in the multidrug efflux system and protein secretion [28,29,40]. After deleting the *sll0180* and *slr2131* genes, which encode the Sll0180 and Slr2131 homologs of AcrA and AcrB in *E. coli*, respectively, *Synechocystis* sp. PCC 6803 mutants had higher intracellular FFA levels but lower extracellular FFA levels [33]. In *E. coli*, the efflux of FFAs and the multidrug efflux system are additionally carried on by the proteins AcrA and AcrB that interact with TolC [41,42].

We highlight the pragmatic results of increased FFA secretion in the cyanobacterium *Synechocystis* sp. PCC 6803 with gene overexpressions of *sll0180* (or *acrA*), *slr2131* (or *acrB*), and *slr1270* (or *tolC*) driven by the *psbA2* promoter generating four strains of OA, OB, OC, and triple OABC, respectively (Table 1 and Figure 1). We verified the overexpression of the gene by comparing the quantity of each transcript to the WTc, even though we did not follow the AcrA, AcrB, or TolC protein levels (Figure 8). Although it is still unclear, we have recently observed the effect of the antibiotic resistance gene in the genome on the FFA secretion in *Synechocystis* during different growth phases. In contrast to the wild-type (WT) strain, which secreted more FFAs amount (%w/DCW) or comparable yield (mg/L) in the late log phase [7,19], the *Synechocystis* sp. PCC 6803 WT control strain (WTc), which carries the *Cm^r^* and/or *Km^r^* gene(s) in its genome, displayed a distinct pattern of decreased FFA secretion from log phase (day 5) to late log phase (day 10) [16]. On the other hand, compared to the WTc, all modified strains did not exhibit the adverse effect on cell growth under normal BG_11_ and BG_11_+1.5% NaCl conditions (Figure 3). Similar to what was plainly seen in the OABC strain, the OB strain slightly lowered its growth around day 10 of culture in BG_11_ medium, which indicated the late-log phase of development (Figure 3A). Although its detrimental impact was not entirely evident, it was speculated that the overexpression of *slr2131* or *acrB* in the OB strain may have contributed to the decrease in cell growth beyond the log stage on day 5 of cultivation. On another note, the growth decrease impact in OB and OABC strains appeared to be mitigated by mild salt stress at a 1.5% NaCl concentration (Figure 3C). On the other hand, we noted that the *sll0180* or *acrA* overexpression in the OA strain enabled cells to grow longer than the WTc under nitrogen-deprived conditions, followed by the native *slr1270* or *tolC* overexpression in the OC and OABC strains (Figure 3B). Furthermore, cyanobacterial cells cultured under nitrogen and required nutrient deficiency experienced the breakdown of photosynthetic pigments, which resulted in chlorosis conditions and a blue-green to yellow color shift in the culture [43,44,45]. According to our findings, all strains underwent a reduction in chlorophyll *a* amounts when nitrogen was depleted (Figure 4B). Interestingly, we further demonstrated that, in comparison to WTc, all overexpressing strains undoubtedly exhibited increased carotenoid levels (Figure 4). Carotenoids, in addition to proteins and lipids, contribute to the bacterial membranes, and they have the ability to regulate the thickness and fluidity of these membranes [46,47,48]. This suggested that modifications to the overproduction of membrane proteins would have an additional impact on carotenoid levels and cellular balance, particularly lipid synthesis, which is a component of the membrane.

The lack of nitrogen could prompt the cells to accumulate their internal biomolecules, such as glycogen, PHB, and lipids, to store energy, maintain their growth, and extend their lives [49,50,51]. When compared to strains under the normal BG_11_ condition, our results showed that intracellular lipid content was significantly enhanced by nitrogen deficiency in all strains (Figure 5B). Nonetheless, in the OB, OC, and OABC strains, the overexpression of the genes *sll2131* (or *acrB*) and *slr1270* (or *tolC*) significantly improved intracellular lipid accumulation, and extracellular FFAs reacted to nitrogen scarcity by 4-, 6-, and 6.7-fold higher than the WTc, respectively (Figure 8). Notably, the overexpressing strains preferred to flow in the direction of fatty acid and lipid synthesis, as shown by their higher content, even though a nitrogen deficit was the source of the PHB accumulation. On the other hand, in *Synechococcus* sp. PCC 7942, salt stress at 250 mM NaCl significantly increased the synthesis of polyunsaturated fatty acids (PUFAs), particularly linoleic acid [52]. Under high concentrations of NaCl at 0.25 M, it demonstrated a remarkable increase in lipid accumulation in freshwater microalgae *Chlamydomonas Mexicana* and *Scenedesmus obliquus* with 37–34% of dry cell weight [53], and in marine algae *Dunaliella* with 67% of dry cell weight under 0.5–1.0 M NaCl [54]. Remarkably, in *Synechocystis* sp. PCC 6803 cells acclimated to 342 mM NaCl, around 57 of the 70 identified proteins were changed. Among them was Slr1270, which is the OprN protein of a multidrug efflux system encoded by the *slr1270* or *tolC* gene [55]. However, in contrast to strains under the normal BG_11_ condition, all overexpressing strains exhibited lower intracellular lipid accumulation during the first five days of cultivation or log stage of growth when exposed to a mild salt stress of 1.5% NaCl concentration. On the other hand, we showed that salt stress increased the expression of the *lipA* transcript, which is implicated in the breakdown of membrane lipids, in all overexpressing strains compared to the WTc by a magnitude of more than 2.7 (Figure 8). It is important to note that the *aas* transcript levels in overexpressing strains were both lower and unaltered in comparison to WTc. This finding may suggest that, in response to salt stress, cells have a comparable or lessened FFA recycling reaction. It may be considered that after counteracting the salt environment, the AcrA, AcrB, and TolC transport system also helps to release certain biomolecules, herein FFAs, into the medium. A recent report addressed the fact that TolC plays a dual role in Gram-negative bacteria, helping to produce exopolysaccharide (EPS) in *Krebsiella pneumoniae* and promoting its antibiotic resistance [56], as well as secreting protein in the cyanobacterium *Synechocystis* sp. PCC 6803 [28]. Furthermore, the loss of Slr1270 (or TolC) function was found necessary for substantial linalool accumulation, suggesting a crucial role in this compound’s secretion in *Synechocystis* sp. PCC 6803 [57]. Our findings then indicate that the AcrA-AcrB-TolC transport pathway in cyanobacteria plays a role in the increased FFA efflux in *Synechocystis* sp. PCC 6803, while further research is required to determine its exact function.

## 4. Materials and Methods

### 4.1. Construction of Recombinant Plasmids

In this study, recombinant plasmids, including pECm_acrA, pECm_acrB, pECm_tolC, and pECm_acrA/acrB/tolC, were constructed (Table 1). A pEERM plasmid containing partial flanking regions of the upstream and downstream DNA sequences of the native *psbA2* gene of *Synechocystis* sp. PCC 6803 wild-type (WT), which has multiple cloning sites (MCS) and chloramphenicol resistance cassette gene (*Cm^r^*) between those sequences. The inserted gene fragments of the *sll0180* (a homolog of *E. coli* acrA, length of 1506 bp), *slr2131* (a homolog of *E. coli* acrB, length of 3300 bp), and *slr1270* (a homolog of *E. coli* tolC, length of 1751 bp) were amplified by PCR using specific pairs of primers (Supplementary Information Table S1), and the genomic DNA of WT was used as the template. The recombinant pECm_acrA plasmid was constructed by ligation of an amplified *sll0180* gene fragment between the restriction sites of *Xba*I and *Spe*I located on the MCS of the pEERM vector [36]. The pECm_acrB plasmid was constructed by the insertion of *slr2131* gene fragment between the restriction sites of *Xba*I and *Spe*I on the vector. The pECm_tolC plasmid was constructed by inserting a *slr1270* fragment between the restriction sites of *Spe*I and *Pst*I on the pEERM vector. The last recombinant pECm_acrA/acrB/tolC plasmid was constructed by sequentially introducing each gene, including *slr2131* and *sll0180* fragments, respectively, into the recombinant pECm_tolC plasmid. All constructions of recombinant plasmids were confirmed by PCR amplification using specific pairs of primers (Supplementary Information Table S1).

### 4.2. Natural Transformation and Confirmation of the Engineered Strains

The host, *Synechocystis* sp. PCC 6803 wild type (WT), was grown in regular BG_11_ medium until it reached an optical density of around 0.3–0.5. The 20 mL cell culture was harvested by centrifugation at 5000 rpm (2516× *g*) for 10 min. The cell pellets were washed with fresh BG_11_ medium once and harvested using centrifugation at 5000 rpm (2516× *g*) for 10 min. For making a condensed cell suspension, 200 µL of new BG_11_ medium was added. A microgram of each recombinant plasmid was then added to the mixture. Subsequently, the mixture was incubated at 28 °C for 6 h, inverting the tubes every 2 h, before spreading on a BG_11_ agar plate containing 35 µg/mL chloramphenicol. Several weeks later, the survival colonies were selected at random and used as templates for PCR analysis using certain primer pairs to validate the location and segregation of the transformants (Supplementary Information Table S2).

### 4.3. Determinations of Cell Growth, Pigment Contents and Oxygen Evolution Rate

All strains were grown in normal BG_11_, BG_11_-N, and BG_11_+1.5% (*w*/*v*, or 256 mM) NaCl conditions with the OD_730_ starter at 0.1 for 16 days. To monitor growth, the cell culture was collected, and the optical density at 730 nm (OD_730_) was measured by using spectrophotometry. One mL of DMF was added to extract the intracellular pigments, including chlorophyll *a* (chl *a*) and carotenoids. The mixture was kept in the dark for 10 min before centrifugation at 6000 rpm (3622× *g*) for 10 min. By measuring the supernatant at absorbances of 425, 625, and 664, respectively, the pigments were identified. The quantities of carotenoid and chlorophyll *a* were then calculated [58,59,60].

At each phase of cell growth, including log (L or day 5) and Late-log (LL or day 10) phases, five mL of cell culture was harvested by centrifugation at 6000 rpm (3622× *g*), 10 min. The cell pellets were washed and resuspended with 5 mL of new BG_11_ medium. After that, the sample suspension was incubated in the darkness for 30 min before determining the oxygen evolution rate with a Clark-type oxygen electrode (Hansatech instruments Ltd., King’s Lynn, UK) at room temperature (25 °C). The unit of O_2_ evolution rate was presented as µmol/mg chlorophyll *a*/h.

### 4.4. Extraction and Determination of Intracellular Lipids and Extracellular FFAs

Five ml of cell culture was centrifuged at 6000 rpm (3622× *g*) for 10 min to separate two fractions, including cell pellets containing the total intracellular lipid and supernatant containing extracellular FFAs. After that, the CHCl_3_:MeOH (ratio 2:1) solvent mixture solution was used to extract both fractions as described previously in [17].

The extracted intracellular lipids and secreted FFAs from cell pellets and supernatant fractions, respectively, were determined using a colorimetric K_2_Cr_2_O_7_ oxidation reaction [61]. A chemical solution (0.5 mL) of 0.18 M K_2_Cr_2_O_7_ and conc. H_2_SO_4_ was added to the extracted lipids and FFAs fractions. The solution mixture was mixed by vortexing and boiled for 30 min to complete the reaction before cooling down to room temperature. Distilled water (0.5 mL) was added and further determined the absorbance at 600 nm by using a spectrophotometer. The commercial canola oil was used as standard and prepared the same protocol as the samples. The contents of total lipids and FFAs were represented as the ratio percentage of lipid weight to dry cell weight (% of dry cell weight or %w/DCW). The dry cell weight (DCW) was obtained by incubating in the oven at 70 °C until the weight of the cells was stable.

### 4.5. Quantitative Analysis of PHB Contents

Ten mL of cell culture was harvested by centrifugation at 6000 rpm (3622× *g*) for 10 min. One hundred µL of 20 mg/mL adipic acid (internal standard) and 800 µL of concentrated H_2_SO_4_ were added to cell pellet fraction. Next, the reaction was boiled at 100 °C for 1 h for hydrolyzing PHB to monomer crotonic acid. Then, 50 µL of the hydrolyzed sample was taken and added into 1.2 mL ultrapure water (UP) for dilution. After that, one mL of each sample was filtered through PP-Syringe filter (0.45 µm, 13 mm) and collected in a glass vial. Then, 10 µL of sample was taken and further injected to HPLC instrument (Shimadzu HPLC LGE System, Kyoto, Japan) using Carbon-18 column with Inert Sustain of 5 µm, 4.6 × 250 mm (UP) (GL-Sciences, Tokyo, Japan) at a flow rate of 1.0 mL/min. The mobile phase was 10 mM KH_2_PO_4_ buffer, pH 2.3, and acetonitrile with a ratio of 70:30 with a UV detector at 210 nm [8,9]. The commercial crotonic acid (Sigma-Aldrich^®^, Inc., St. Louis, MO, USA) was used as standard and prepared as same as the sample. The contents of PHB were represented as the ratio percentage of PHB weight to dry cell weight (%w/DCW).

### 4.6. Determination of Transcription Levels by Reverse Transcription Polymerase Chain Reaction (RT-PCR)

Twenty mL of the cell culture at log phase of cell growth (or day 5) under normal BG_11_, BG11-N, and BG_11_+1.5% NaCl conditions was harvested by centrifugation at 6000 rpm (3622× *g*) for 10 min. The total RNAs were extracted by adding 1 mL of TRIzol^®^ Reagent (Invitrogen, Life Technologies Corporation, Carlsbad, CA, USA). Then, the total RNA extract was treated with RNaseI-free DNAseI (Fermentas, Carlsbad, CA, USA) to remove the DNA contamination before cDNA synthesis. A ReverTra Ace^®^ qPCR RT Master Mix (TOYOBO Co., Ltd., Osaka, Japan) was used to synthesize cDNA. The transcription level of genes, including *sll0180*, *slr2131*, *slr1270*, *lipA*, *aas*, *plsX*, *crtB*, *chlG*, and *16s* rRNA, was determined via an RT-PCR analysis using cDNA as the template and the specific pairs of primers (Supplementary Information Table S1). The PCR condition and Tm of each gene were addressed in Supplementary Information Table S2. KOD polymerase was used in PCR amplification. The PCR conditions were 98 °C for 3 min, followed by proper cycles of each gene (Supplementary Information Tables S1 and S2) at 98 °C for 10 s, the primer melting temperature (Tm) for 10 s, 68 °C for 10 s to extend the DNA strand, and 68 °C for 3 min at the last step. The PCR products were verified by 1.5% (*w*/*v*) agarose gel electrophoresis before quantifying by AmershamTM ImageQuantTM 800 gel documentation instrument (GE Healthcare Life Sciences, Marlborough, MA, USA).

### 4.7. Statistical Analysis

The results of the two experiments were compared using the Microsoft Excel software Version 16.85. The two-paired sample t-test was the statistical method employed. A risk threshold of *p* = 0.05 was used for all statistical analyses, and a value of *p* < 0.05 was considered statistically significant.

## 5. Conclusions

Under N-deprivation and NaCl stress conditions, engineered strains of *Synechocystis* sp. PCC 6803 with *acrAB-tolC* overexpression exhibited increased amounts of internal lipids and extracellular free fatty acids. In Figure 8, a summary of products and transcript levels of all overexpressing (OX) strains compared with the WTc is shown under normal (BG_11_), nitrogen deprivation (BG_11_-N), and salt stress (BG_11_+15% NaCl) at day 5 of treatment. The enhanced accumulation of intracellular lipids and the remarkable increase in extracellular FFAs were both significantly impacted by the nitrogen-deprived state. The most significant potential for FFA efflux in *Synechocystis* sp. PCC 6803 was evidently addressed by the triple overexpression of the *sll0180_slr2131_slr1270* genes. All overexpressing strains exhibit increased carotenoid accumulation, which has been suggested to be associated with lipid synthesis in the context of altered membrane homeostasis under stress. Additional research investigating the useful role of the AcrAB-TolC transport system in cyanobacteria might yield a potential benefit for future biotechnological applications and large-scale manufacturing.

## Figures and Tables

**Table 1:** Strains and plasmids used in this study.

Name	Relevant Genotype	Reference
Cyanobacterial strains		
*Synechocystis* sp. PCC 6803	Wild type	Pasteur culture collection
WTc	*Cm^r^* integrated at the native *psbA2* gene in *Synechocystis* WT genome	[16]
OA	*sll0180*, and *Cm^r^* integrated at the native *psbA2* gene in *Synechocystis* WT genome	This study
OB	*slr2131*, and *Cm^r^* integrated at the native *psbA2* gene in *Synechocystis* WT genome	This study
OC	*slr1270*, and *Cm^r^* integrated at the native *psbA2* gene in *Synechocystis* WT genome	This study
OABC	*sll0180*, *slr2131*, *slr1270* and *Cm^r^* integrated at the native *psbA2* gene in *Synechocystis* WT genome	This study
Plasmids		
pEERM	P*psbA2–Cm^r^*; plasmid containing *Cm^r^* between the flanking region of upstream and downstream *psbA2* sequences	[36]
pECm_acrA	P*psbA2*–*sll0108*-*Cm^r^*; plasmid containing *sll0108* and *Cm^r^* between the flanking region of upstream and downstream *psbA2* sequences	This study
pECm_acrB	P*psbA2*–*slr2131-Cm^r^*; plasmid containing *slr2131* and *Cm^r^* between the flanking region of upstream and downstream *psbA2* sequences	This study
pECm_tolC	P*psbA2*–*slr1270-Cm^r^*; plasmid containing *slr1270* and *Cm^r^* between the flanking region of upstream and downstream *psbA2* sequences	This study
pECm_acrA/acrB/tolC	P*psbA2*–*sll0108-slr2131-slr1270-Cm^r^*; plasmid containing *sll0108, slr2131, slr1270*, and *Cm^r^* between the flanking region of upstream and downstream *psbA2* sequences	This study

P*psbA2*, strong *psbA2* promoter; *Cm^r^*, chloramphenicol resistance cassette.

**Table 2:** Yields (mg/L) of intracellular lipid and extracellular FFAs of *Synechocystis* sp. PCC 6803 WTc, OA, OB, OC, and OABC strains cultured in BG_11_, BG_11_-N, and BG_11_+1.5% NaCl (*w*/*v*) media. Data represent mean ± S.D., *n* = 3. The statistical difference between the values of WTc and engineered strain under each treatment time is represented by an asterisk, * *p* < 0.05.

Strain(s)	Intracellular Lipid Yield (mg/L)		Extracellular Lipid Yield (mg/L)	
	Start		Start	
WTc	17.1 ± 1.7		n.d.	
OA	17.7 ± 1.8		n.d.	
OB	16.3 ± 2.2		n.d.	
OC	16.5 ± 1.1		n.d.	
OABC	16.0 ± 0.1		n.d.	
Normal BG_11_	Day 5	Day 10	Day 5	Day 10
WTc	82.4 ± 4.1	171.4 ± 11.8	69.3 ± 9.1	84.3 ± 3.7
OA	73.7 ± 2.0 *	172.4 ± 12.5	107.7 ± 14.6 *	69.5 ± 8.6 *
OB	77.0 ± 7.8	173.8 ± 11.3	112.6 ± 20.7 *	68.3 ± 3.0 *
OC	78.2 ± 5.2	170.7 ± 13.0	126.2 ± 9.9 *	83.6 ± 15.5
OABC	94.1 ± 6.9 *	170.7 ± 11.3	47.1 ± 12.7 *	80.7 ± 2.5
BG_11_-N	Day 5	Day 10	Day 5	Day 10
WTc	30.1 ± 3.1	27.6 ± 1.0	27.6 ± 5.4	75.2 ± 6.5
OA	22.7 ± 4.0 *	28.4 ± 3.1	62.1 ± 8.1 *	85.0 ± 10.7
OB	32.1 ± 1.8	27.1 ± 2.1	82.6 ± 2.9 *	90.0 ± 6.2 *
OC	27.1 ± 1.8	26.9 ± 3.6	101.9 ± 9.3 *	103.1 ± 8.4 *
OABC	27.1 ± 2.0	28.3 ± 3.0	99.8 ± 8.5 *	84.5 ± 6.2 *
BG_11_+1.5% NaCl	Day 5	Day 10	Day 5	Day 10
WTc	76.2 ± 3.6	108.3 ± 11.4	215.5 ± 15.8	168.1 ± 6.4
OA	91.0 ± 4.4 *	126.4 ± 10.5	303.8 ± 4.4 *	155.2 ± 16.9
OB	81.9 ± 10.3	132.6 ± 12.2 *	308.3 ± 31.3 *	136.4 ± 19.6 *
OC	81.4 ± 7.4	139.1 ± 8.3 *	268.6 ± 18.8 *	153.8 ± 2.5 *
OABC	105.2 ± 5.8 *	103.6 ± 5.0	269.3 ± 16.3 *	138.8 ± 15.9 *

n.d., undetectable.

## Data Availability

Data are contained within the article and Supplementary Materials.

## Supplementary Materials

The following supporting information can be downloaded at: https://www.mdpi.com/article/10.3390/ijms252212131/s1.

## Abbreviations

AAS	acyl–acyl carrier protein synthetase
ACP	acyl carrier protein
Car	carotenoids
Chl *a*	chlorophyll *a*
CO_2_	carbon dioxide
DCW	dry cell weight
DMF	*N*,*N*-dimethylformamide
FFA	free fatty acid
GGPP	geranylgeranyl pyrophosphate
h	hour
IM	inner membrane
lipA	lipase A
m	meter
μg	microgram
mL	milliliter
min	minute
nm	nanometer
OD	optical density
OM	outer membrane
PCR	polymerase chain reaction
plsX	putative acyltransferase
PHB	polyhydroxybutyrate
rpm	revolutions per minute
s	seconds
S-layer	surface layer protein
WT	wild type
