# Supplementary material for: Triple-Gene Overexpression of the AcrA-AcrB-TolC Transporter System in Synechocystis sp. PCC 6803 Contributes to a Higher Secretion of Free Fatty Acids in Response to Nitrogen Shortage and Salt Stress
Source: Int J Mol Sci. 2024 Nov 12;25(22):12131. doi: 10.3390/ijms252212131 (PMC11594277; doi:10.3390/ijms252212131)
Supplement: Supplementary file 1 [file ijms-25-12131-s001.zip › ijms-3251606-supplementary.pdf]

Table S1. Primers used in this study.

Name	Sequence (5' to 3')	Purpose of primer	Expected size (bp)	Cycles/Tm	Reference
Sll0180_F	AGACAATCTAGAATGGTTAGAAAACGCTCC	PCR for *sll0180* (*acrA*)	1,506	30 /59.0 °C	This study
Sll0180_R	AGACAAACTAGTTTACAGGGAAGACTCGAG	PCR for *sll0180* (*acrA*)			This study
Slr2131_F	AGACAATCTAGAGAGTCGGCTGGAGATTGAGCC	PCR for *slr2131* (*acrB*)	3,300	30 /59.0 °C	This study
Slr2131_R	AGACAAACTAGTCTAGCTATTCCTTTGGGA	PCR for *slr2131* (*acrB*)			
Slr2170_F	AGACAAACTAGTGGTTGGAGATCCCCGGCGAT	PCR for *slr1270* (*tolC*)	1,751	30 /56.0 °C	This study
Slr2170_R	AGACAACTGCAGCCCATCGTCTCAGAC	PCR for *slr1270* (*tolC*)			This study
UUSpsbA2_F	CACTCAGATAGGAGCCATCTTGC	Colony PCR			Eungrasamee et al. 2020
DDSpsbA2_R	CACTCAGATAGGAGCCATCTTGC	Colony PCR			Eungrasamee et al. 2020
Cm_R	CAGCTCGAGGCTTGGATTCT	Colony PCR			Eungrasamee et al. 2020
RTacrA_F360	CAACCTAGTGGCTACCAACT	RT-PCR for *sll0180*	360	28 /54.0 °C (BG_{11})	This study
RTacrA_R360	GCCAAGTTAAGATTGGCCTG	RT-PCR for *sll0180*		32 /54.0 °C (BG_{11}-N)	
				28 /54.0 °C (BG_{11}+ 1.5%NaCl)	
RTacrB_F380	CAATGCGGAAGTGGTGGAAA	RT-PCR for *slr2131*	380	28 /54.0 °C (BG_{11})	This study
RTacrB_R380	CAAAAATCCGTACATCCCCC	RT-PCR for *slr2131*		32 /54.0 °C (BG_{11}-N)	
				28 /54.0 °C (BG_{11}+ 1.5%NaCl)	
RTtolC_F480	CTGAGAAATAATGAACAACTCCAGC	RT-PCR for *slr1270*	480	30 /56.0 °C (BG_{11})	This study
RTtolC_R480	CCAACCCGGCCTGTTCTAAC	RT-PCR for *slr1270*		33 /56.0 °C (BG_{11}-N)	
				30 /56.0 °C (BG_{11}+ 1.5%NaCl)	
RTlipA_F379	TTGGCGGAGCAAGTGAAGCAAT	RT-PCR for *lipA*	379	30 /56.0 °C (BG_{11})	Eungrasamee et al. 2019
RTlipA_R379	CATGGACCAGCACAGGCAAAAT	RT-PCR for *lipA*		33 /56.0 °C (BG_{11}-N)	
				30 /56.0 °C (BG_{11}+ 1.5%NaCl)	
RTplsX_F488	AAGGGGTGGTGGAAATGGAA	RT-PCR for *plsX*	488	33 /52.0 °C (BG_{11})	Towijit et al. 2018
RTplsX_R488	AAGTAGGTCCCTTCCTTCGG	RT-PCR for *plsX*		39 /52.0 °C (BG_{11}-N)	
				32 /52.0 °C (BG_{11}+ 1.5%NaCl)	

**Name**	**Sequence (5' to 3')**	**Purpose of primer**	**Expected size**	**Cycles/Tm**	**Reference**
RTaas_F307	GTGGTTTATCGCCGATCAAG	RT-PCR for *aas*	307	33 /55.0 °C (BG_{11})	Eungrasamee et al. 2019
RTaas_R307	TTCCTGGCGGGGAACGGGAG	RT-PCR for *aas*		32 /55.0 °C (BG_{11}-N)	
				32 /55.0 °C (BG_{11}+1.5%NaCl)	
RTcrtB_F462	TGACCAATATTCTGCGGGACGTT	RT-PCR for *crtB*	462	33 /56.0 °C (BG_{11})	Natesungnoen et al. 2023
RTcrtB_R462	TAGAGACTGCAGAGTGAGTCCTTAGC	RT-PCR for *crtB*		30 /56.0 °C (BG_{11}-N)	
				29 /56.0 °C (BG_{11}+ 1.5%NaCl)	
RTchlG_F435	TACATTGCCCTACCGTGGTGG	RT-PCR for *chlG*	435	30 /59.0 °C (BG_{11})	Natesungnoen et al. 2023
RTchlG_R435	TCAAATCCCCGCATGGCCTA	RT-PCR for *chlG*		32 /59.0 °C (BG_{11}-N)	
				30 /59.0 °C (BG_{11}+1.5%NaCl)	
RT16SrRNA_F521	AGTTCTGACGGTACCTGATGA	RT-PCR for *16S*	521	15 /56.0 °C (BG_{11})	Natesungnoen et al. 2023
RT16SrRNA_R521	GTCAAGCCTTGGTAAGGTTAT	RT-PCR for 16S		19 /56.0 °C (BG_{11}-N)	
				15 /56.0 °C (BG_{11}+1.5%NaCl)	

Table S2 PCR conditions for checking transformants

Strains	Pair of primers	Cycles/Tm	Expected size (bp)
OA	UUSpsbA2/ DDSpsbA2<br>Sll0180_F /Cm_R	30 /56.0 °C<br>30 /59.0 °C	3966<br>2506
OB	UUSpsbA2/ DDSpsbA2<br>Slr2131_F /Cm_R	30 /56.0 °C<br>30 /57.0 °C	5760<br>4,300
OC	UUSpsbA2/ DDSpsbA2<br>Slr2170_F /Cm_R	30 /56.0 °C<br>30 /58.0 °C	4211<br>2751
OABC	Sll0180_F / RTacrB_R380<br>Sll0180_F / RTtolC_R480<br>UUSpsbA2/ Sll0180_R<br>Slr2131_F /Cm_R	30 /59.0 °C<br>30 /56.0 °C<br>30 /57.0 °C<br>30 /57.0 °C	2158<br>5766<br>2176<br>6051

Figure S1: The expression of genes in each strain cultured under normal BG_{11} medium analyzed by RT-PCR analysis

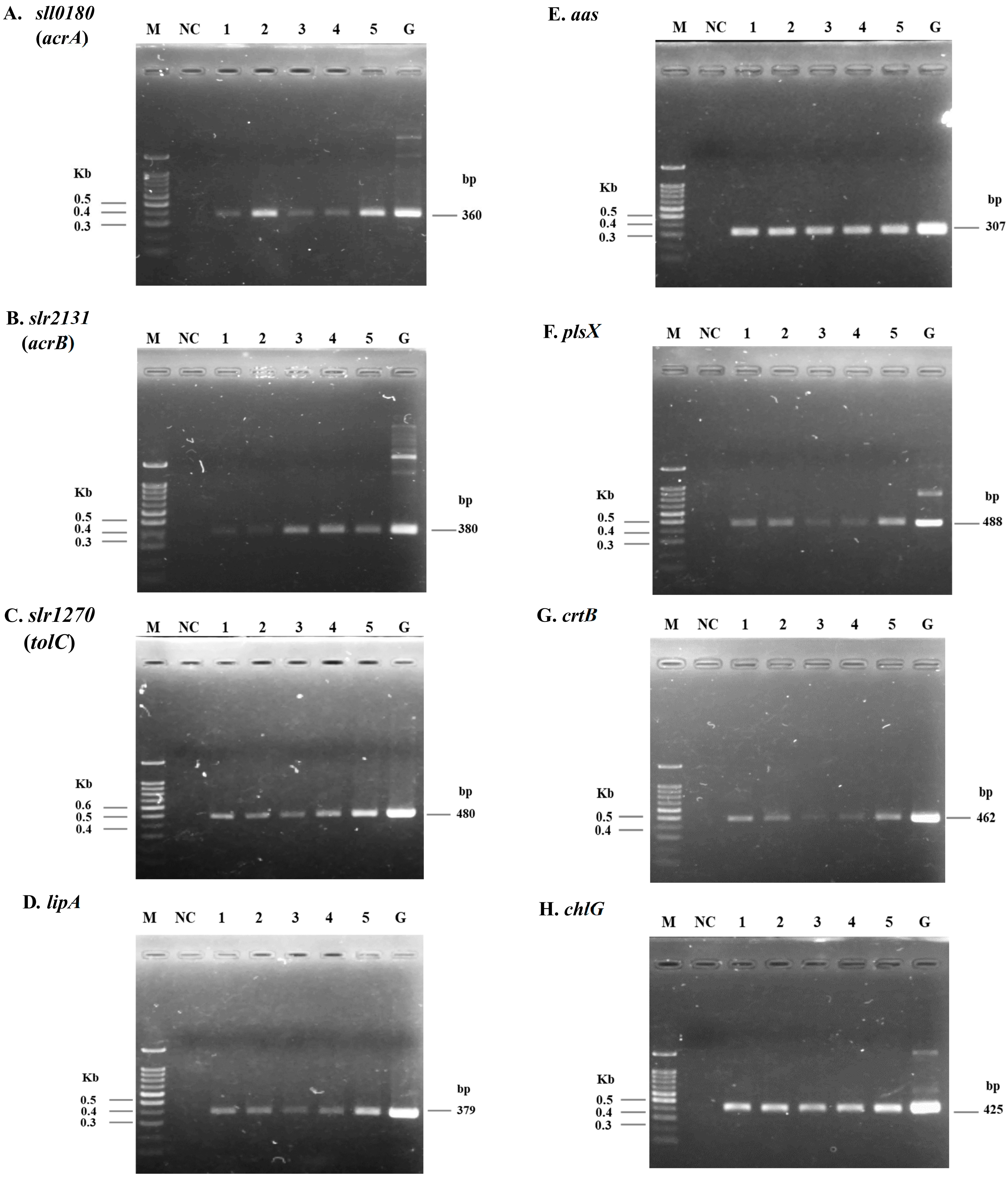

Figure S1 (Continue): The expression of genes in each strain cultured under normal BG_{11} medium analyzed by RT-PCR analysis

I. ***16S r*****RNA**

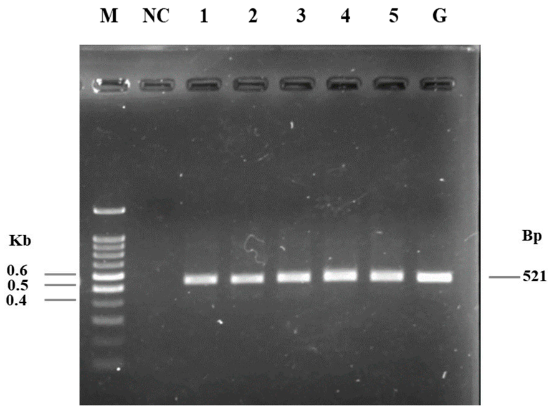

Figure legend S1. The figure displays images of 1% agarose electrophoresis gels stained by RedSafe™ Nucleic acid staining. The products were obtained from the RT-PCR analysis of all strains that were grown in normal BG_{11} medium. The expression levels of nine genes were analyzed using specific primer pairs (listed in Table S2). The genes analyzed were A. *sll0180* (360 bp), B. *slr2131* (380 bp), C. *slr1270* (480 bp), D. *lipA* (379 bp), E. *aas* (307 bp), F. *plsX* (455 bp), G. *crtB* (462 bp), H. *chlG* (425 bp), and I. *16s r*RNA (521 bp). The gel electrophoresis included; Lane M: M23 Set of 100 bp + 1,5 Kb DNA ladder with stain, SibEnzyme®; Lane NC: Negative control, Lane 1-5: PCR products from studied cells, including WTc, OA, OB, OC, and OABC, respectively; and Lane G: PCR product of genomic DNA of *Synecocystis* sp PCC 6803 wild type.

Figure S2: The expression of genes in each strain cultured in BG_{11} - N medium analyzed by RT-PCR analysis

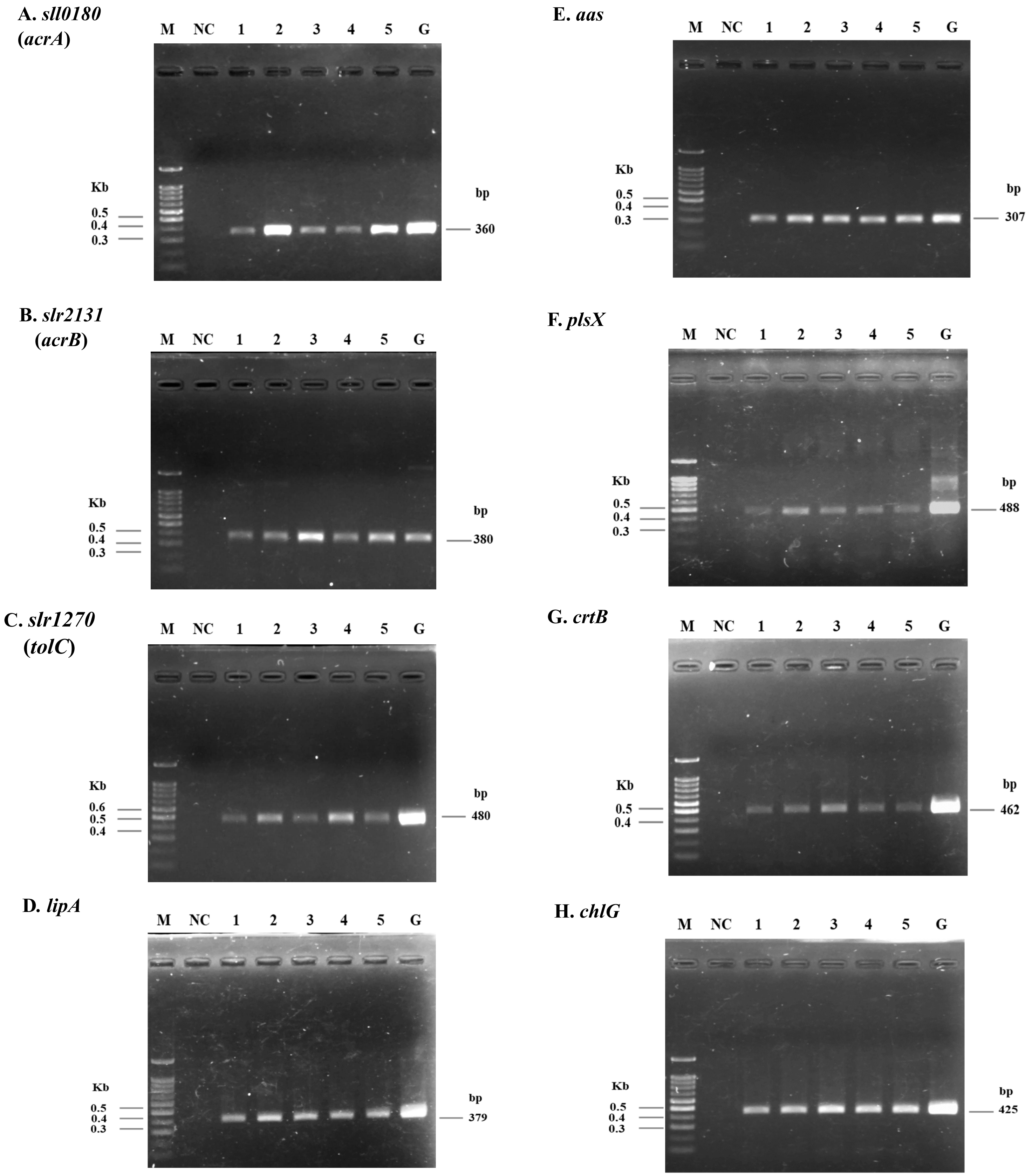

Figure S2 (Continue): The expression of genes in each strain cultured in BG_{11} - N medium analyzed by RT-PCR analysis

I. *16S r*RNA

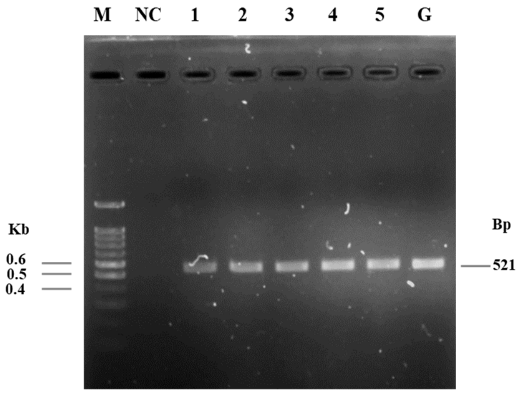

Figure legend S2. The figure displays images of 1% agarose electrophoresis gels stained by RedSafe™ Nucleic acid staining. The products were obtained from the RT-PCR analysis of all strains that were grown in BG_{11} -N medium. The expression levels of nine genes were analyzed using specific primer pairs (listed in Table S2). The genes analyzed were A. *sll0180* (360 bp), B. *slr2131* (380 bp), C. *slr1270* (480 bp), D. *lipA* (379 bp), E. *aas* (307 bp), F. *plsX* (455 bp), G. *crtB* (462 bp), H. *chlG* (425 bp), and I. *16s r*RNA (521 bp). The gel electrophoresis included; Lane M: M23 Set of 100 bp + 1,5 Kb DNA ladder with stain, SibEnzyme®; Lane NC: Negative control, Lane 1-5: PCR products from studied cells, including WTc, OA, OB, OC, and OABC, respectively; and Lane G: PCR product of genomic DNA of *Synecocystis* sp PCC 6803 wild type.

Figure S3: The expression of genes in each strain cultured in BG_{11}+1.5% NaCl medium analyzed by RT-PCR analysis

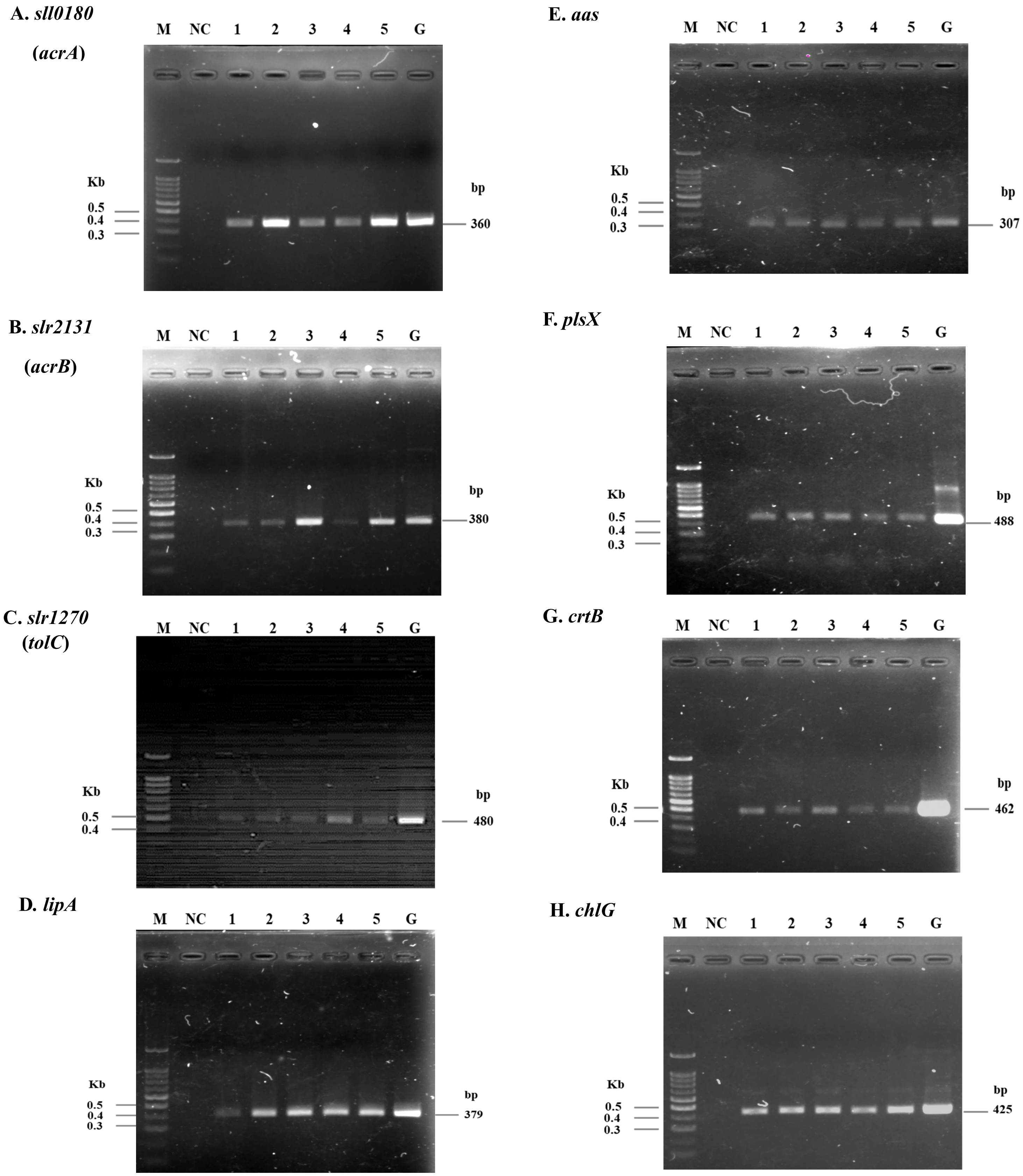

Figure S3 (Continue): The expression of genes in each strain cultured in BG_{11} +1.5% NaCl medium analyzed by RT-PCR analysis

I. *16S r*RNA

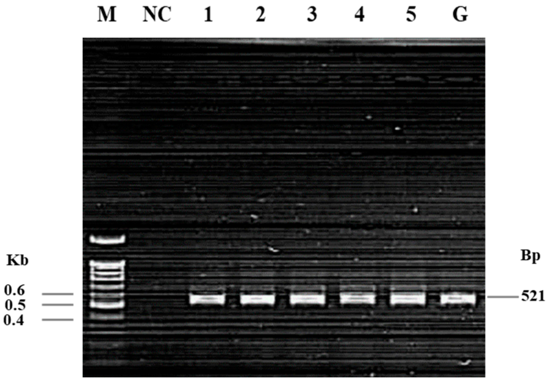

Figure legend S3. The figure displays images of 1% agarose electrophoresis gels stained by RedSafe™ Nucleic acid staining. The products were obtained from the RT-PCR analysis of all strains that were grown in BG_{11} + 1.5% NaCl medium. The expression levels of nine genes were analyzed using specific primer pairs (listed in Table S2). The genes analyzed were A. *sll0180* (360 bp), B. *slr2131* (380 bp), C. *slr1270* (480 bp), D. *lipA* (379 bp), E. *aas* (307 bp), F. *plsX* (455 bp), G. *crtB* (462 bp), H. *chlG* (425 bp), and I. *16s r*RNA (521 bp). The gel electrophoresis included; Lane M: M23 Set of 100 bp + 1,5 Kb DNA ladder with stain, SibEnzyme®; Lane NC: Negative control, Lane 1-5: PCR products from studied cells, including WTc, OA, OB, OC, and OABC, respectively; and Lane G: PCR product of genomic DNA of *Synecocystis* sp PCC 6803 wild type.
